# Arteannuin-B and (3-Chlorophenyl)-2-Spiroisoxazoline Derivative Exhibit Anti-Inflammatory Effects in LPS-Activated RAW 264.7 Macrophages and BALB/c Mice-Induced Proinflammatory Responses via Downregulation of NF-κB/P38 MAPK Signaling

**DOI:** 10.3390/molecules27228068

**Published:** 2022-11-20

**Authors:** Gifty Sawhney, Javeed Ur Rasool, Diksha Saroch, Mumin Ozturk, Frank Brombacher, Bilal Ahmad, Asha Bhagat, Asif Ali, Suraj P. Parihar, Zabeer Ahmed

**Affiliations:** 1 Pharmacology Division, CSIR-Indian Institute of Integrative Medicine, Canal Road, Jammu 180001, India; 2 Academy of Scientific and Innovative Research (AcSIR), Ghaziabad 201002, India; 3 Natural Product and Medicinal Chemistry Division, CSIR-Indian Institute of Integrative Medicine, Canal Road, Jammu 180001, India; 4 International Centre for Genetic Engineering and Biotechnology (ICGEB), Cape Town 7925, South Africa; 5 Wellcome Centre for Infectious Diseases Research in Africa (CIDRI-Africa), Institute of Infectious Diseases and Molecular Medicine (IDM), Department of Pathology, Faculty of Health Sciences, University of Cape Town, Cape Town 7700, South Africa; 6 Department of Molecular Science and Technology, Ajou University, Suwon 16499, Republic of Korea; 7 CSIR-Institute of Genomics and Integrative Biology, Sukhdev Vihar, New Delhi 110025, India; 8 Department of Biochemistry, Human Metabolomics, Faculty of Natural and Agricultural Sciences, North-West University, Potchefstroom 2531, South Africa

**Keywords:** Arteannuin-B, spirocyclic-2-isoxazoline derivative, inflammation, BALB/c mice, TNF-α, IL-6, NF-κB, iNOS, LPS, cytokines, RAW 264.7 cells

## Abstract

Host inflammatory responses are key to protection against injury; however, persistent inflammation is detrimental and contributes to morbidity and mortality. Herein, we demonstrated the anti-inflammatory role of Arteannuin-B (**1**) and its new spirocyclic-2-isoxazoline derivative **JR-9** and their side effects in acute inflammatory condition in vivo using LPS-induced cytokines assay, carrageenan-induced paw edema, acetic acid-induced writhing and tail immersion. The results show that the spirocyclic-2-isoxazoline derivative is a potent anti-inflammatory agent with minimal cell toxicity as compared to Arteannuin-B. In addition, the efficacies of these compounds were also validated by flow cytometric, computational, and histopathological analysis. Our results show that the anti-inflammatory response of **JR-9** significantly reduces the ability of mouse macrophages to produce NO, TNF-α, and IL-6 following LPS stimulation. Therefore, JR-9 is a prospective candidate for the development of anti-inflammatory drugs and its molecular mechanism is likely related to the regulation of NF-κB and MAPK signaling pathway.

## 1. Introduction

Inflammation is characterized by the activation of immune and nonimmune cells that guard the host organism against viral and bacterial invasion to promote tissue repair and regeneration. However, chronic inflammation is detrimental to the host if it goes unchecked [1,2,3,4,5,6,7,8]. Inflammatory cells are associated with a broad range of physical and mental health issues that control morbidity and mortality due to ischemic heart disease, diabetes, renal infections, nonalcoholic fatty liver disease (NAFLD), and neurodegenerative disorders. Therefore, an urgent need for innovative anti-inflammatory medication exists due to the resistance, toxicity, and side effects of the current drugs in use.

The proinflammatory cytokines, such as interleukins (IL-6) and tumor necrosis factor-α (TNF-α) and nitric oxide synthase (iNOS), are mediators of inflammatory mechanisms [9,10]. In this context, the activation of the transcription factor NF-κB has been well-established to mediate these inflammatory responses [11,12,13,14,15,16,17]. Toll-like receptors (TLRs), expressed on macrophages, cause pathogenic bacteria to induce NF-κB upon stimulation [18,19]. Conversely, IkB inhibitors of NF-κB prevent the activation of transcription factors in the cytoplasm of naive cells [20,21,22,23,24]. The activation of NF-κB is done by IkB, which phosphorylates IKK, thus causing NF-κB to become active. Therefore, the enzyme and cytokine activities in pro- and anti-inflammatory processes delicately balance the immune system’s operation [25,26,27,28]. However, NF-κB controls the production of inflammatory mediators like iNOS, TNF-α, and IL-6, which not only aid in the elimination of pathogens but also, if in excess, cause inflammatory damage as in rheumatoid arthritis and septic shock [29]. Phosphorylation of mitogen-activated protein kinases (MAPKs) like p38-MAPK and extracellular signal-regulated kinase (ERK) is a crucial step for the production of proinflammatory cytokines in activated macrophages [21]. Therefore, significant investigations have been performed to find safe and potent anti-inflammatory candidates that could involve the expression of inflammatory proteins via upstream signaling mechanisms.

**Figure 1:**
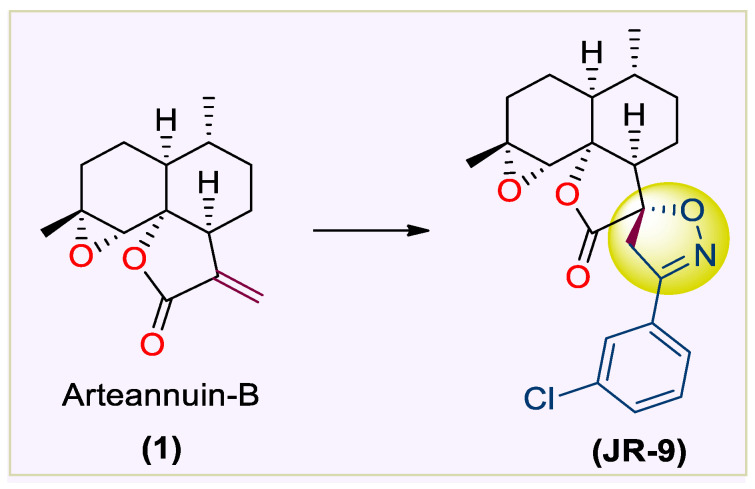
Structure of **1** and **JR-9** (Rasool et al. 2021).

On the other hand, heterocyclics, like substituted spirocyclic-2-isoxazolines, are the structural building block of many biologically active scaffolds and molecular leads in inflammation drug discovery [30,31,32,33,34]. The therapeutic efficacy of **1** has been investigated in recent years for its antineoplastic activity in vitro [35,36], apoptosis [36,37], antileukaemia [37,38] and antiproliferative activity in breast, lung, cervical, and liver cancer cells [38]. In our previous study [39]., we reported the synthesis and anti-inflammatory screening of spiro-isoxazoline adducts of **1,** which furnished the **JR-9** derivative as a potent compound with lower toxicity [22] (Figure 1).

After achieving the encouraging results from the previous study, herein we report the anti-inflammatory and antianalgesic potentials of **1** and **JR-9** in BALB/c mice and murine macrophages, which could be effective for developing a lead anti-inflammatory drug candidate.

## 2. Results

### 2.1. Arteannuin-B (**1**) and **JR-9** Had No Cytotoxic Effect on Macrophages

**Figure 2:**
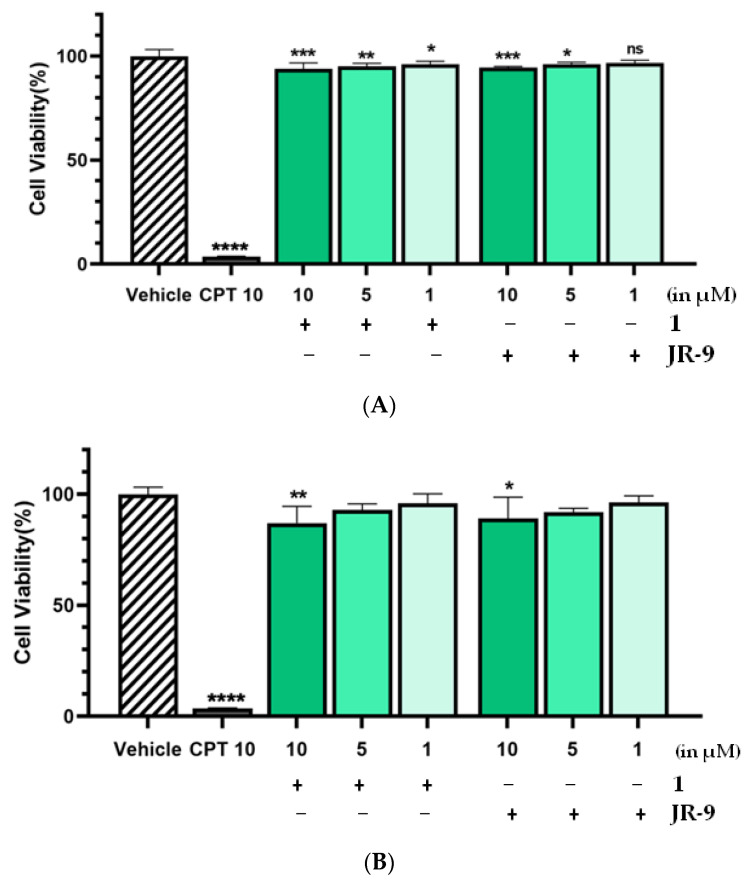
The cells were treated with different concentrations of **1** and **JR-9**, and the cell viability was determined by MTT assay after 24 h and 48 h treatment in Figure 2A and Figure 2B, respectively. The results shown are the mean ± standard deviation of three independent experiments. Statistical significance was assessed by one-way ANOVA followed by Dunnett’s test, **** *p* < 0.0001, *** *p* < 0.001, ** *p* < 0.005, * *p* < 0.05 vs. vehicle. All the values were mean ± SD, *n* = 4. CPT: Camptothecin.

The cell viability of RAW 264.7 remained unaffected even at the higher dose (10 µM) of **1** and **JR-9** (Figure 2). At 24 h experiment, the cell viability exhibited by **1** and **JR-9** was found to be 93.96% and 94.55%, respectively, showing no cytotoxicity.

Therefore, the pharmacological effects of the **1** and **JR-9** can be evaluated at higher, noncytotoxic dosages in 48 h experiments, which resulted in 87.1% and 89.05% viability, respectively. Our results revealed that at 1–10 μM, **1** and **JR-9** are nontoxic and thus may be used to explore the anti-inflammatory effect on RAW 264.7 macrophages.

### 2.2. Arteannuin-B (**1**) and **JR-9** Inhibited the Production of NO, TNF-α and IL-6 in Macrophages

**Figure 3:**
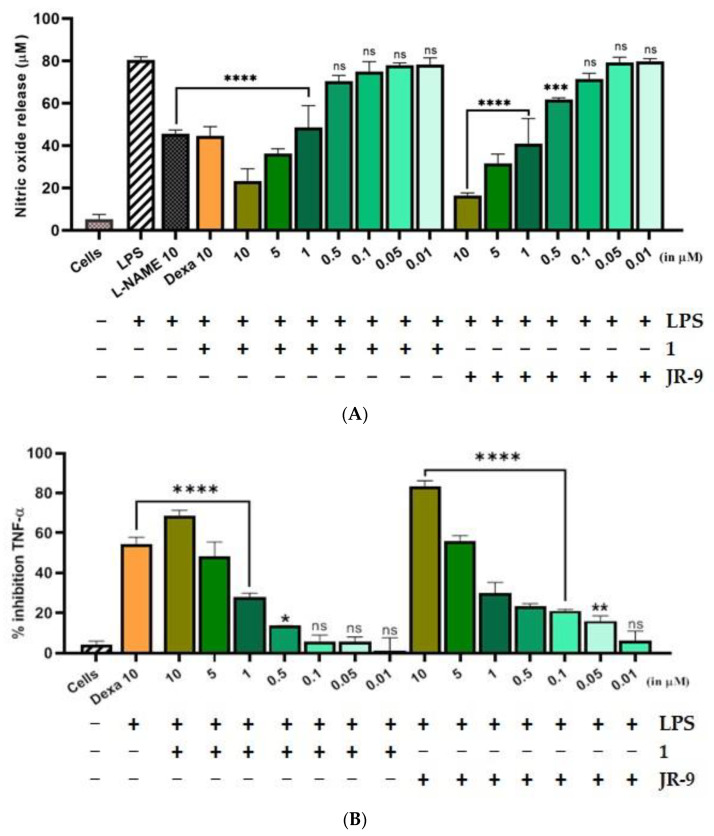

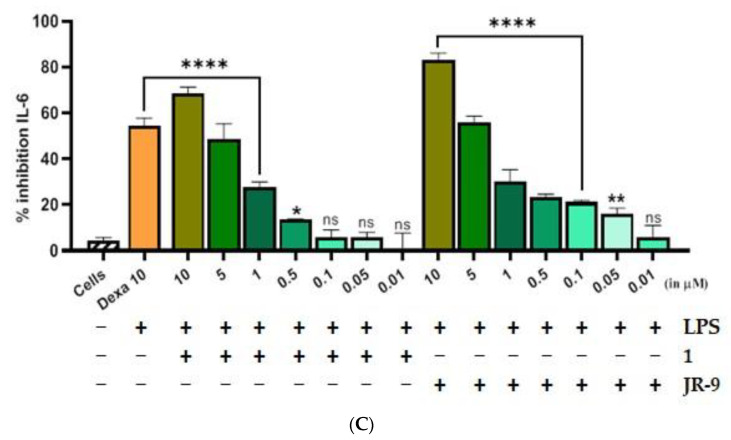
Comparative effect of **1** and **JR-9** on (**A**) NO, (**B**) TNF-α, and (**C**) IL-6 release in LPS stimulated RAW 264.7 cells. The cells were treated with different concentrations of **1** and **JR-9** for 1 h, and LPS (1 µg/mL) was then added for 24 h. The levels of nitric oxide in the cell culture supernatant were measured using the Griess reagent assay, and the levels of TNF-α and IL-6 in the cell culture supernatant were measured using ELISA. All values were mean ± SD, *n* = 3. (**** *p* < 0.0001, *** *p* < 0.0005, ** *p* < 0.005, * *p* < 0.05) indicated a significant difference with the LPS-treated cells assessed by one-way ANOVA followed by Dunnett’s test. L-NAME: L-NG-Nitro arginine methyl ester; LPS: lipopolysaccharide; Dexa: dexamethasone; TNF-α: tumor necrosis factor-alpha; IL-6: interleukin-6.

We further explored whether **1** and **JR-9** have the ability to reduce the proinflammatory mediator, nitric oxide (NO), and proinflammatory cytokines TNF-α and IL-6 produced by the macrophages. As compared to L-NAME, **1** and **JR-9** suppressed LPS-stimulated nitrite levels in a dose-dependent manner (Figure 3A). L-NAME and Dexamethasone (10 µM) were used as positive controls and suppressed the LPS-induced levels of nitric oxide from 80.38 µM to 45.61 µM and 44.5 µM, respectively. While as **1** suppresses the release of NO to 23.29 µM at 10 μM, 36.26 µM at 5 µM, 48.65 µM at 1 µM; **JR-9**, on the other hand, suppresses the same to 16.43 µM at 10 µM, 31.62 µM at 5 µM, 40.97 µM at 1 µM, proving **JR-9** to be the better nitric oxide inhibitor as compared to **1 [40]**.

The percentage inhibition of TNF-α and IL-6 due to compound **1** was found to be (72.18%) and (68.43%) at 10 μM, while the percentage inhibition of the same cytokines due to **JR-9** was found more effective for TNF-α (81.58%) and IL-6 (83.34%) at the same dose. Dexa was used as the positive control, which exhibited percentage inhibition of 61.18% and 54.56% for TNF-α and IL-6, respectively (Figure 3B). In the case of IL-6, **JR-9** exhibited a higher percentage inhibition of 55.9% compared with 48.5% inhibition of **1** at 5 μM and 54.5% Dexa (10 μM). These results indicate that **JR-9** efficiently inhibits LPS-induced proinflammatory cytokine production as compared to **1** in RAW 264.7 macrophages (Figure 3C).

### 2.3. Arteannuin-B (**1**) and **JR-9** Inhibit the Production of Reactive Oxygen Species (ROS) in Macrophages

**Figure 4:**
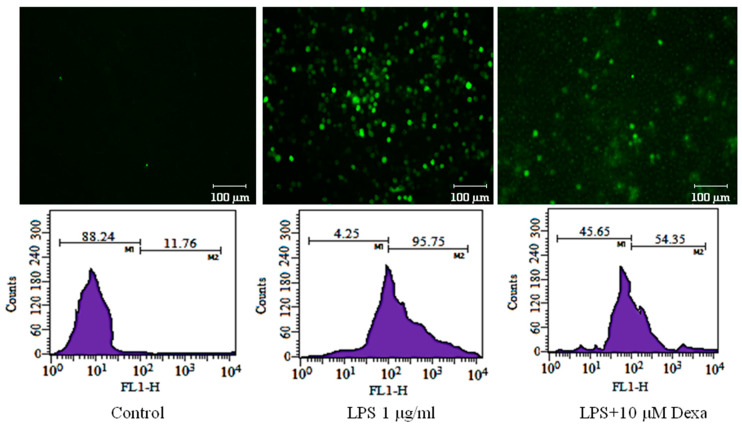

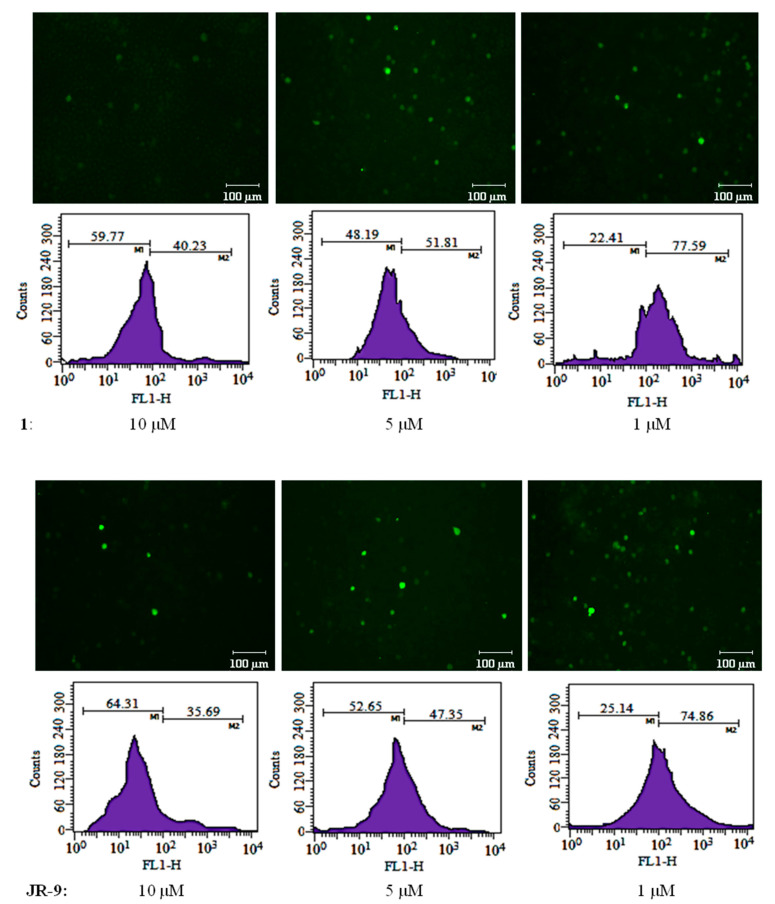
To determine the effect of **1** and **JR-9** on the intracellular ROS production, treated RAW 264.7 cells were incubated with DCFH-DA (10 mM) for 30 min. The relative fluorescence intensity of fluorophore DCFH-DA was then measured and detected using BD FACS Aria III flow cytometer (488 nm excitation, 530–540 nm emission), respectively. The results shown are the mean ± standard deviation of three independent experiments.

During inflammation, the activated macrophages produce a substantial quantity of ROS [41,42,43]. Intracellular ROS in LPS-stimulated RAW 264.7 cells show a 95.75% increase as compared to 11.76% in untreated cells. The pretreatment of **JR-9,** as compared to **1,** reduced ROS from 40.23%, 51.81%, 77.59% to 35.69%, 47.35%, 74.86% at 10 µM, 5 µM, and 1 µM, respectively, whereas the dexamethasone reduced ROS to 54.35% at 10µM (Figure 4).

### 2.4. Arteannuin-B (**1**) and **JR-9** Interact with the DNA Binding Site of NF-κB

**Figure 5:**
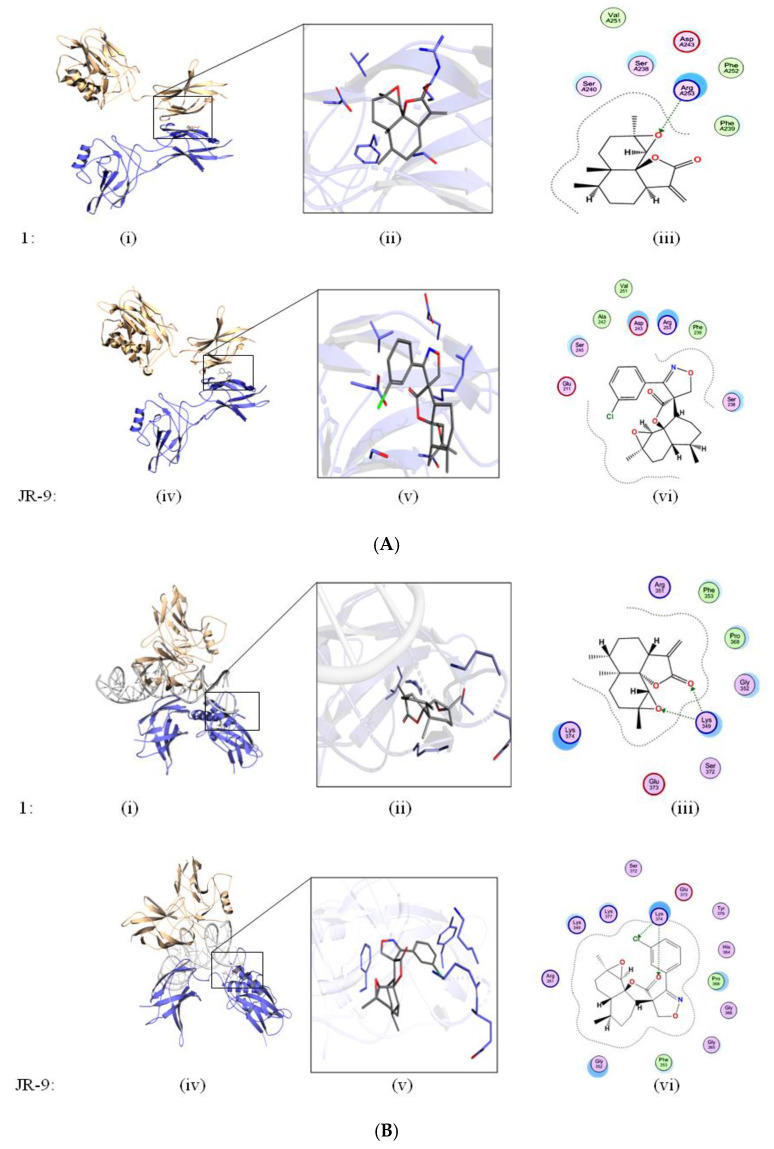
(**A**) Ligands docked at Dimeric site. In silico studies to determine the binding interaction of **1** and **JR-9** with ligands docked at dimeric interface site. The molecular operating environment (MOE2019) was used to assess the behavior of **1** and **JR-9** in the binding site of NF-κB. Green lines show the iconic interaction of the molecule with that of different amino acids. The interacting residues within the 4.5 Å of the ligand are shown in 2D diagram. (**B**) Ligands docked at DNA-binding site. In silico studies to determine the binding interaction of **1** and **JR-9** with ligands docked at DNA binding site. The molecular operating environment (MOE2019) was used to assess the behavior of **1** and **JR-9** in the binding site of NF-κB. Green lines show the iconic interaction of the molecule with that of different amino acids.

Molecular docking was performed to see the binding efficiency of the ligands (**1** and **JR-9**) with the target protein (NF-κB) at two different sites. The dimeric interface and DNA binding sites (Figure 5A,B) were selected as suitable sites. **JR-9** showed interaction with Glu211, Asp243, Arg253, Phe239, and Ser238 (Figure 5(A,vi)) at the dimeric interface, while **1** showed similar interactions with the same amino acids at the dimeric interface site (Figure 5(A,iii)). Analyzing the interaction of the docked solution at the DNA binding site (Figure 5B), the **JR-9** showed ionic interaction with Lys374 and arene type π–π interaction with Tyr379 (Figure 5(B,vi)). In addition to these interactions, **JR-9** engages with Arg351, Lys349, Lys377, His364, Gly366, Gly365, and Phe353 (Figure 5(B,vi)). Similarly, **1** interacts with Arg351, Phe353, Gly352, Lys349, and Lys374 (Figure 5(B,iii)). **1** showed the ionic interaction with Lys349 of NF-κB.

The binding affinity of **1** and **JR-9** was computed by Cyscore analysis, as shown in Table 1. The docking score and binding affinity data showed that both molecules scored higher than the dimeric site. The docking and binding affinity data revealed that **1** and **JR-9** have comparative affinities towards the DNA binding site of NF-κB. Further, we evaluate the ADME properties of these compounds by utilizing SwissADME (Lausanne, Switzerland). The ADME data (Table 1) showed that both molecules have the same pharmacokinetic and drug-likeness properties. **1** and **JR-9** compounds were docked on NF-κB at the dimeric interface site and DNA interacting site. The docked solutions were clustered, and the most populated cluster was selected from which top representative members’ docking score, Cyscore score, and ADME properties are mentioned in Table 1.

### 2.5. Arteannuin-B (**1**) and **JR-9** Inhibited NF-κB and MAPK Activation

**Figure 6:**
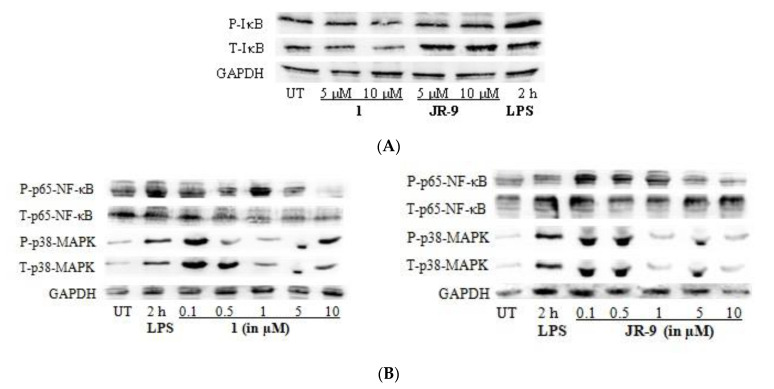
(**A**) Impact of **1** and **JR-9** on the P-IκB and T-IκB pathway in RAW 264.7 cells. LPS-induced RAW 264.7 cells were treated with **1** and **JR-9** for 2 h in different concentrations. The NF-κB and MAPK signaling pathway expression proteins were detected using Western blotting. (**B**) Impact of **1** and **JR-9** on the NF-κB and MAPK pathway in RAW 264.7 cells. LPS-induced RAW 264.7 cells were treated with **1** and **JR-9** for 2 h in different concentrations. The NF-κB and MAPK signaling pathway expression proteins were detected using Western blotting.

The regulation of inflammatory mediators in LPS-stimulated macrophages is transcriptionally linked with the NF-κB and MAPK pathways [26,44]; the effects of **1** and **JR-9** on LPS-induced degradation and phosphorylation of inhibitory kappa B (IκB) protein were examined using immunoblotting. At 5 µM and 10 µM, compounds **1** and **JR-9** blocked LPS-induced phosphorylation of IκB in the cytosol in a dose-dependent manner (Figure 6A). When IκB-α and NF-κB are separated, free dimer-activated subunits of NF-κB (p50/p65) can be translocated from the cytosol to the nucleus.

LPS alone increased the quantity of NF-κB in the nucleus, whereas **1** and **JR-9** reduced LPS-induced nuclear translocation of NF-κB in a dose-dependent manner [45] (Figure 6B). We determined the effect of **1** and **JR-9** on LPS-stimulated phosphorylation of P-p38 MAPK and T-p38 MAPK in macrophages to determine whether the inhibition of NF-κB activation is mediated through MAPK pathways. At 2 h, LPS significantly promoted the phosphorylation of p38, whereas LPS-stimulated phosphorylation of p38 MAPK was considerably reduced by pretreatment with **1** and **JR-9**. These findings suggest that, compared to MAPK, NF-κB may have a role in **1** and **JR-9** ability to reduce NO and proinflammatory cytokines in activated macrophages.

### 2.6. Arteannuin-B (**1**) and **JR-9** Are Safe in Acute Toxicity

In the acute toxicity study, the oral LD50 of **1** and **JR-9** was >2000 mg/kg. The doses for **1** and **JR-9** were calculated according to the LPS-induced dose-response experiment with six doses of 50, 40, 20, 10, 5, and 1 mg/kg. Beyond 40 mg/kg concentration, the percentage inhibition was found to be less in the LPS-induced dose-response experiment for TNF-α and IL-6 and carrageenan-induced paw edema model. Therefore, for the current investigation, we chose the highest dose, 40 mg/kg and found no deaths, toxicity symptoms, or behavioral abnormalities, suggesting that **1** and **JR-9** are reasonably safe at 40 mg/kg concentration.

### 2.7. Arteannuin-B (**1**) and **JR-9** Reduced the Production of TNF-α and IL-6 in the Serum in LPS-Treated Mice

**Figure 7:**
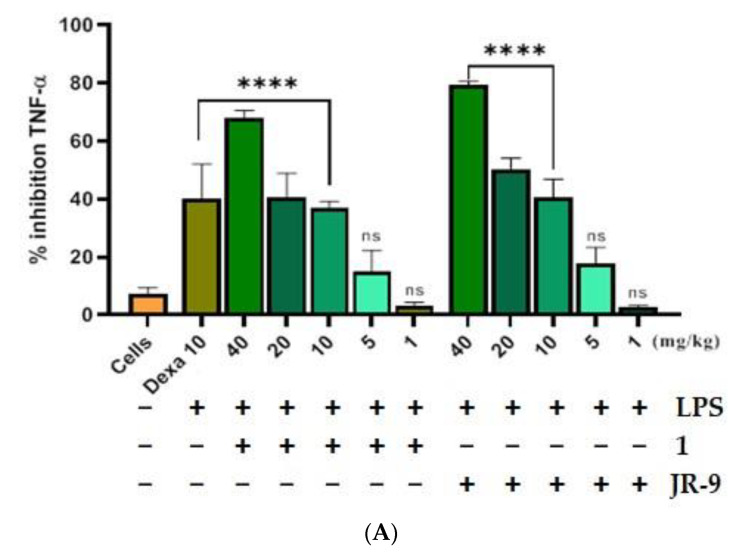

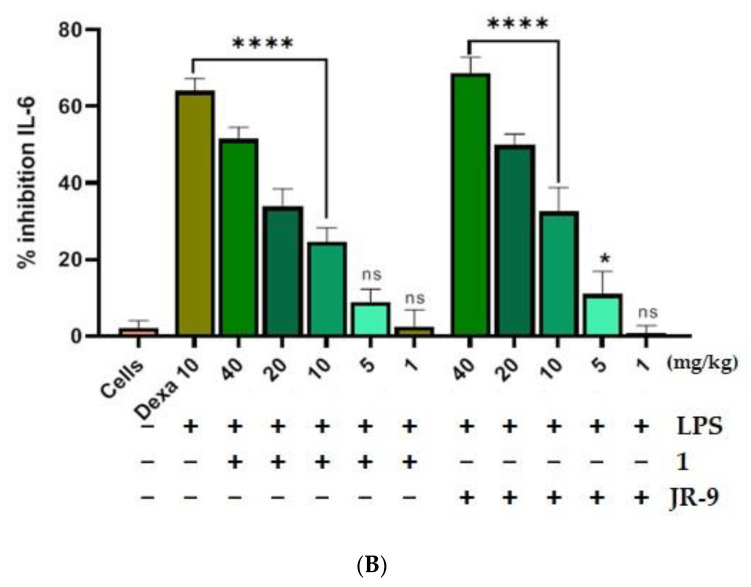
Comparative effect of **1** and **JR-9** on proinflammatory cytokines in LPS (1 mg/kg)-treated mice. **1** and **JR-9** at 40, 20, 10, 5, and 1 mg/kg were given orally, blood was collected from ROP, and cytokine (**A**) TNF-α and (**B**) IL-6 inhibition was measured in serum. All values were mean ± SD, *n* = 4. (**** *p* < 0.0001, * *p* < 0.05, ns = non-significant) indicated significant difference with the LPS treated mice assessed by one-way ANOVA followed by Dunnett’s test). LPS: lipopolysaccharide; Dexa: dexamethasone; TNF-α: tumor necrosis factor alpha; IL-6: interleukin-6.

We first assessed the outcome of LPS-induced septic shock in mice treated with **1** and **JR-9**. LPS injection to the intraperitoneal cavity of mice releases proinflammatory cytokines like TNF-α and IL-6 in the blood, which were then quantified from the serum using ELISA. The **JR-9** (40, 20, 10, 5, 1 mg/kg, p.o.) was found to inhibit TNF-α by 79.42%, 50.37%, 40.76%, 17.64%, 2.85% and IL-6 by 68.6%, 49.75%, 32.75%, 11%, 3.25%, as compared to TNF-α inhibition of **1** by 67.96%, 40.74%, 37.08%, 15.02%, 2.29%, and IL-6 by 51.4%, 33.8%, 24.6%, 9.3%, and 2.4%, respectively. The standard drug, dexamethasone at 10 mg/kg, showed 40.14% TNF-α and 64.05% IL-6 inhibition (Figure 7).

### 2.8. JR-9 Efficiently Reduced Writhing in Mice Compared to Arteannuin-B (1)

**1** and **JR-9** showed a dose-dependent reduction in the number of writhes in mice compared to the Diclofenac. **1** and **JR-9**, at the doses of 40, 20, 10, 5, and 1 mg/kg p.o., reduced the writhing significantly in a dose-dependent manner by 53.03%, 43.74%, 23.97%, 10.94%, 7.48%, and 60.21%, 53.29%, 33.05%, 23.01%, and 8.25%, respectively, in response to nociceptive pain induced by acetic acid in i.p. cavity of mice. Treatment with Diclofenac reduced writhing by 40.42% at a 20 mg/kg dose. The results of the writhing syndrome model are shown in Table 2.

### 2.9. **JR-9** Increased the Reaction Time in Mice Compared to Arteannuin-B (**1**)

The tail immersion test investigated the effect of the analgesic medications, which act on the central nervous system by increasing the reaction time of mice in response to thermal stimulus induced by hot water. As shown in Table 3, **1** and **JR-9** had a strong analgesic effect from 30 min onwards, with the maximum effect occurring 120 min after treatment. The impact was statistically significant compared to the mice in the control group.

### 2.10. JR-9 Decreased the Paw Edema Induced by Carrageenan in Mice Compared to Arteannuin-B (1)

**Figure 8:**
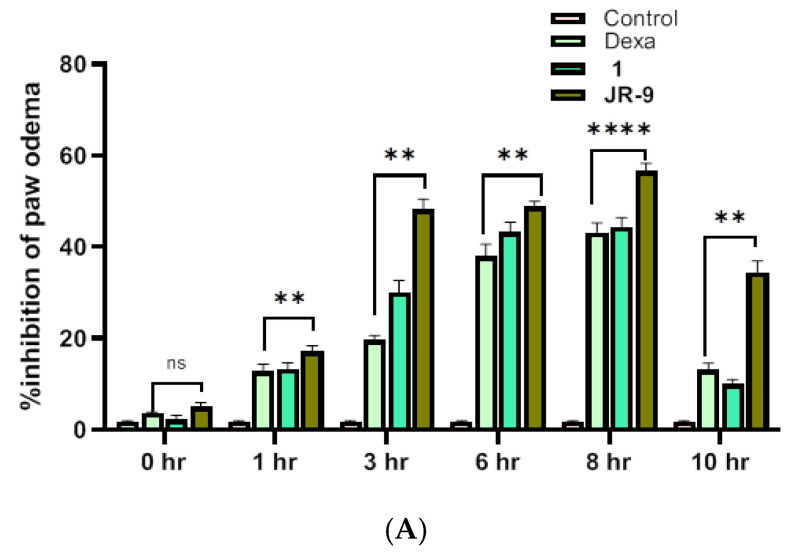

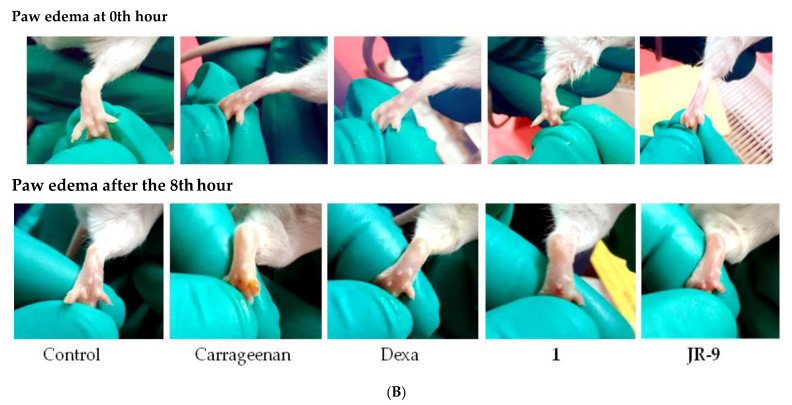
(**A**) Graphs showing an increase in the inhibitory effect of **JR-9** compared to **1** and Dexa (20 mg/kg); the maximum effect was noticed at the 8th hour of the subcutaneous injection of carrageenan. Each value represents mean ± SD, *n* = 5 (**** *p* < 0.0001, ** *p* < 0.05 indicated significant difference as compared to the control group assessed by two-way ANOVA followed by Tukey test). (**B**) Comparative effect of **1** and **JR-9** at 40 mg/kg dose on carrageenan-induced paw edema: Compounds or vehicle was administrated 1 h prior to carrageenan injection (1%), and paw volume measured.

Subcutaneous injection of carrageenan increases the paw size by eliciting edema, which is a marker of acute paw inflammation in mice. Figure 8A shows the inhibitory effect of **1** and **JR-9** (40 mg/kg) with 44.4% and 56% inhibition, respectively, compared to dexamethasone (20 mg/kg) with 43.15% at 8th hr. Images are representative of each group of animals treated as indicated (Figure 8B). The determination of paw edema revealed a significant reduction in **1** and **JR-9** treated groups compared to the control group of animals. 

### 2.11. Arteannuin-B (**1**) and **JR-9** Did Not Affect Biochemical and Haematological Analysis in Mice

The serum biochemical analysis revealed no consistent changes between the treatment and control groups (Table 4). Throughout the study, the readouts such as HCT, MCV, WBC, and RBC counts remain in the normal range in treated and control animals. However, there were occasional alterations in neutrophils, lymphocyte, and monocyte counts in treated groups and control groups. The result suggests that **1** and **JR-9** had no major effects on the biochemical and hematology of animals.

### 2.12. Arteannuin-B (**1**) and **JR-9** Inhibit Tissue Pathology of Mice

To investigate the effects of **1** and **JR-9** on different organ tissues, we assessed the histopathological changes of each group by Hematoxylin-Eosin staining. LPS induces systemic inflammatory responses and injuries on kidney, liver and lung tissues during sepsis.

Several histopathological changes in kidney were observed after LPS treatment. Various parts of the kidney, including renal corpuscles, tubules, blood vessels, and interstitium in the cortex, medulla, and papilla, were examined. Renal epithelial tubular cells were sloughed and decreased epithelial cells in kidney. However, there was no evidence of pathological effects in **1** and **JR-9** group.

Similar to kidney injury, LPS-induced liver injury showed significant necrosis. The hepatic morphology and architecture were normal in the control group. Compared to the control group, the LPS group showed obvious inflammatory cell infiltration, cell spotty necrosis, and nuclear pyknosis fragmentation, demonstrating severe septic liver injury which diminished after administration of **1** and **JR-9.**

In LPS group, Hematoxylin-Eosin stained lungs showed increased thickness of alveolar wall and inflammatory cell infiltration and the control group showed no pathological changes. Pathological changes observed in the BALB/c mice treated with **1** and **JR-9** showed weaker changes as compared to the mice treated with LPS. In the microscopic evaluation of the lungs, all the muscular layers and air sacs were examined to reveal no evidence of tissue pathology.

**Figure 9:**
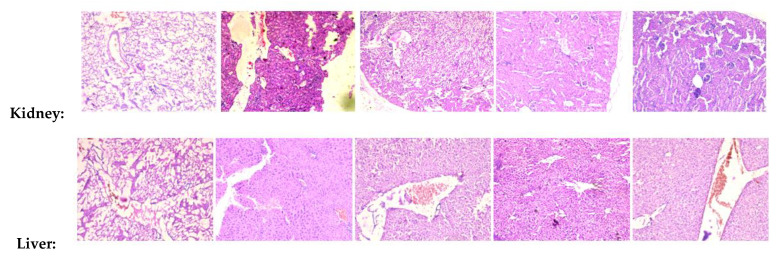

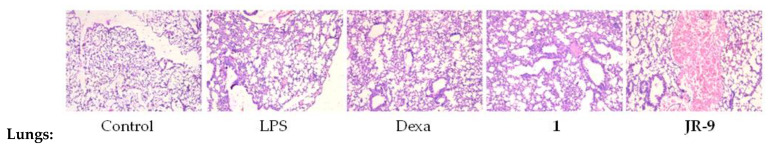
Histopathological analysis of kidney, liver, and lung tissues. BALB/c mice were injected with **1** and **JR-9** (40 mg/kg) 1 h before LPS injection (1 mg/kg i.p), and kidney, liver, and lung tissues were harvested 24 h after LPS injection. **JR-9** (40 mg/kg) protects the liver, kidney, and lungs from more than 1 (40 mg/kg) damage. These results depict H&E staining of a liver tissue section from the indicated group (40× magnification).

Interestingly, treatment with **1** and **JR-9** remarkably treated the injury induced by LPS. The microscopic examination of different tissues showed no significant pathological changes. In the test groups of animals, no toxic or toxic-allergic effects of **1** and **JR-9** were found (Figure 9).

## 3. Discussion

The vast majority of newly discovered medicines are based on the structural variety of natural compounds which have the potential to interact with a variety of biological targets commonly. These compounds can either be found in their natural or semi-synthetic derivatives. NSAIDs, for example, Dexamethasone and Diclofenac, are, globally, the most prescribed medications in clinical and veterinary sciences for managing inflammatory disorders [46,47]. Despite the clinical efficacy of these drugs, there is a substantial risk of adverse responses like myocardial infarction, liver and kidney damage, allergic reactions and gastrointestinal bleeding [48]. 25% of patients with such ailments typically have adverse effects, with 5% of cases that result in imminent death. This necessitates the quest for more substantial and safer analgesic and anti-inflammatory drugs.

Keeping this purview in mind, we investigated the anti-inflammatory potential of **JR-9** compared to its parent molecule **1**. Initially, the library of 14 compounds was screened, which furnished 03 hits **JR-9**, JR-11, and JR-14. Out of these hits, (3-chlorophenyl)-2-spiroisoxazoline (**JR-9**) did not affect cell survival or shape and significantly reduced the inflammatory responses in LPS-stimulated macrophages. Interestingly, treatment of BALB/c mice and RAW 264.7 cells with (**JR-9**) significantly outperformed dexamethasone in suppressing NO equivalent to that of its parent molecule **1** by inhibiting LPS-induced NF-κB activation from controlling the production of iNOS.

In addition, the regulation of NF-κB is essential for developing proinflammatory cytokines TNF-α and IL-6. In order to confirm the production of TNF-α and IL-6, ELISA was performed, which indicate that **1** and **JR-9** have a potential inhibitory effect upon these cytokines.

Microscopic and flow cytometric analysis of intracellular ROS levels showed that pre-treatment with **JR-9** efficiently reduced the excessive ROS production caused by LPS-stimulated RAW 264.7 cells [49]. Similarly, our results demonstrate that **JR-9** is a potential suppressor of LPS-induced degradation of IκBα and NF-κB p65 nuclear translocation factor. Our studies reveal that LPS induces the NF-κB/IκBα pathway, which stimulates the production of proinflammatory cytokines.

Molecular docking studies show that the binding efficiency of ligand **1** and **JR-9** at the DNA site of the target protein NF-κB, respectively, is more (BE = −2.53 and −2.87 kcal/mol) as compared with that at the dimeric site (BE = −2.34 and −2.54 kcal/mol). Moreover, histopathological study shows no adverse effect of the **1** and **JR-9** concentrations employed in an animal model; nevertheless, mice treated with carrageenan showed the build-up of infiltrating inflammatory cells, vascular congestion, and negative necrotic response. The biochemical and hematological toxicity indicators also did not exhibit any abnormalities.

Since the primary issue brought on by inflammation is pain; therefore, we performed the tail immersion test in order to study the increase in reaction time in response to thermally stimulated pain for analgesia/nociceptive activity. In this test, we observed an increase in the reaction time in response to thermally produced pain which could, perhaps, indicate the antianalgesic property of **JR-9**. Similarly, the outcome of the carrageenan-induced edema and the acetic acid-induced writhing demonstrated the potency of the **JR-9** derivative against chemically generated pain.

**JR-9**, the potent anti-inflammatory and analgesic semisynthetic compound with a comprehensive safety window, showed an almost higher potential than the most popular NSAIDs. Even **JR-9** is administered at a low dose of 1 mg/kg, indicating its therapeutic potential. Therefore, **JR-9** can be a suitable candidate for further exploration of its therapeutic potential against chronic-inflammatory models and detailed mechanistic studies for unravelling the mode of action.

## 4. Materials and Methods

### 4.1. Chemicals

Streptomycin (100 ng/mL) and penicillin (100 U/mL) were purchased from Sigma (Gillingham, UK); 96 well plates were provided by NUNC, (Havel, Germany), while GIBCO (Grand island, NY) provided fetal bovine serum (FBS). DMSO, dexamethasone, lipopolysaccharides (LPS—Escherichia coli O111:B4), *N*-nitro-l-arginine methyl ester (L-NAME), Griess reagent (1% sulfanilamide/0.1% naphthyl ethylenediamine dihydrochloride in 2.5% H_3_PO_4_), camptothecin, 3-(4,5-dimethylthiazol-2-yl)-2,5-diphenyltetrazolium bromide (MTT) were bought from Sigma, IL-6 and TNF-α ELISA Kits were procured from Invitrogen (Massachusetts, US). Synergy Mx microplate reader belonged to BioTek, Winooski, VT, USA. P-IκB, T-IκB, P-p65-NF-κB, T-p65-NF-κB, P-p38-MAPK, and T-p38-MAPK, GAPDH, and anti-rabbit IgG horseradish peroxidase (HRP) conjugated secondary antibodies were purchased from Cell Signaling Technology (Danvers, MA, USA).

### 4.2. Cell Culture

RAW 264.7 macrophages were procured from ATCC (Rockville, MD, USA), and were grown in GIBCO’s Dulbecco’s Modified Eagle Medium (DMEM) supplemented with 10% fetal bovine serum (FBS), 100 U/mL penicillin, and 100 ng/mL streptomycin, and incubated at 37 °C in a 5% CO_2_ environment. For all the experiments, the cells were used between the third and fourth passages.

### 4.3. MTT Assay

RAW 264.7 macrophages were seeded into 96 well plates (20,000 cells per well) for 24 h to assess the viability. The cells were treated with **1** and **JR-9** for 48 h, camptothecin (positive control), or vehicle control (0.1% DMSO) before being combined with 20 µL of MTT solution (final concentration of 0.25 mg/mL). After 4 h of incubation, the supernatant was removed, the formazan crystals were dissolved in 100 µL DMSO, and absorbance at 570 nm was measured with a Synergy Mx microplate reader (BioTek, Winooski, VT, USA) [50]. The average of quadruplicate determinations with the mean standard error was used to illustrate the findings (SD). The cells’ survival was determined using the formula below:

$$cell\;viability\;\left(\%\right)=\frac{\left(absorbance\;of\;treated\;cells\right)}{\left(absorbance\;of\;untreated\;cells\right)}\times 100$$

### 4.4. Measurement of Nitrite by Griess Reagent

RAW 264.7 macrophages were seeded at a density of 1 × 10^5^ cells per well in 96-well plates. After 24 h of incubation, these cells were treated with 1 µg/mL LPS and different concentrations of **1** and **JR-9**. The nitrite (NO^2−^) level in the culture medium was used to evaluate the effectiveness of NO release. This was performed by mixing an equal ratio of the supernatant with the Griess reagent (1:1). After 10 min of incubation at room temperature, optical density was determined at 540 nm. The NO concentration was quantified using the with the LPS stimulated group’s nitrate level serving as the positive control. Final calculations were based on a NaNO_2_ standard curve made with linear concentrations of sodium nitrate.

### 4.5. Cytokine Production

RAW 264.7 cells were seeded at a density of 2 × 10^5^ cells/well in 96-well plates. After an overnight incubation, the cells were treated with **1**, **JR-9** and dexamethasone (10 µM) was used as a positive control. After one hour of treatment, 1µg/mL LPS was added and incubated for 24 h. After 24 h, the cell culture supernatants were collected, and TNF-α and IL-6 levels were determined using ELISA Kits (Invitrogen) according to the manufacturer’s instructions. A standard curve was used to quantify the concentrations of TNF-α and IL-6 in the samples. An equation was used to compute the percent inhibition, displayed below. The data is the average of three independent determinations with a standard mean error (SD).

$$cytokine\;inhibition\;\left(\%\right)=\left[\frac{Control-Treated}{Control}\right]\times 100$$

### 4.6. Measurement of Intracellular Reactive Oxygen Species

RAW 264.7 cells were plated in a six-well culture plate, at the concentration of 1 × 10^6^ cells per well and treated with 1 µg/mL LPS for 24 h and different concentrations of **1** and **JR-9**. The cells were washed twice in 1× PBS to eliminate the extracellular debris. After 30 min of incubation with 10 mM DCFH-DA at 37 °C, the cells were washed twice with 1× PBS. The DCFH-DA fluorescent probe was used to measure intracellular ROS DCFH-DA oxidized to the highly fluorescent chemical dichlorofluorescein by intracellular H_2_O_2_ or low-molecular-weight peroxides (DCF). Flow cytometry and a fluorescent microscope (Nikon Corporation, Chiyoda-ku, Tokyo, Japan) were used to investigate ROS generation in the cells (BD FACS Aria III, 488 nm excitation, 530–540 nm emission). A minimum of 20,000 events were examined in each sample, with the results presented as a fold-change in fluorescence intensity over time.

### 4.7. Molecular Docking and Cyscore Analysis

**1** and **JR-9** were docked on NF-κB (Protein databank (PDB) ID: 3GUT) by employing the Molecular Operating Environment (MOE) 2019.01 docking program (Chemical Computing Group ULC, Montreal, QC, Canada). The NF-κB PDB structure was retrieved from the RCBS protein database. The breaks in the structure were repaired. The partial charges were added to the proteins after water molecules were removed and hydrogen atoms were added. MOE2019.01 was used to reduce the protein architectures utilizing the Optimized potential for liquid simulations (OPLS) force field. In ChemDraw, the 3-dimensional structure of **1** and **JR-9** was created, and the MMFF94x force field was used to reduce the structure in **MOE2019.01**. After protein and ligand preparation, the ligands **1** and **JR-9** were docked on NF-κB at the dimeric interface and DNA binding sites. The docked solutions were clustered based on root mean square deviation and docking score. Representative members of the populated cluster were analyzed for binding affinity by cys-score analysis [51]. In addition, we evaluated the ADME properties of **1** and **JR-9** by utilizing SwissADME [52].

### 4.8. Western Blot Analysis

RAW 264.7 macrophage cells (2 × 10^5^ cells/well) were plated in a 6-well plate and treated for 24 h with or without **1** and **JR-9** (5–10 μM), followed by the treatment of LPS (1 µg/mL) for 2 h. The cells were rinsed in ice-cold 1× PBS (1 mL, pH 7.4) and lysed in RIPA buffer. Thermo Scientific Pierce^TM^, Massachusetts, USA. B.C.A. Protein Assay Kit was used to quantify the protein concentration. 10% SDS-polyacrylamide gel was used to separate 25 μg of proteins, which were then transferred to nitrocellulose membranes (NC) followed by immunoblotting. The membranes were incubated overnight with primary antibodies to identify the target proteins specific for IκB, p65 NF-κB, p38 MAPK and GAPDH at 4 °C. After three 5-min washes with TBS-T solution, the membrane was incubated for 1 h at room temperature with HRP-linked anti-mouse, anti-rabbit, or anti-goat immunoglobulin G secondary antibodies [53,54]. The detection was performed using enhanced chemiluminescence (ECL), ChemiDOC (Davinch-K, Davinch-Invivotm) imaging system.

### 4.9. Animals

All the animal studies followed the guidelines in the Guide for the Care and Use of Laboratory Animals (National Research Council 2011). The protocols employed in the experiment were authorized by the Institutional Animal Ethics Committee of CSIR-Indian Institute of Integrative Medicine (180/75/8/2019). One week before the start of the study, specific pathogen-free BALB/c mice (female, 6–8 weeks old, 20–25 g) were kept under standard laboratory conditions: 23 ± 1 °C, 55 ± 10% relative humidity, 12/12 h light/dark cycles, with filtered tap water and standard pellet food (Lipton India Ltd.). Body weight was measured regularly to adjust the dosing volumes. Blood was collected from the retro-orbital plexus in heparinized tubes and centrifuged at 10,000 rpm for 5 min. The serum was separated and stored at −80 °C until use for cytokines detection. Biochemical and hematological parameters were investigated at the end of the study, and the animals were sacrificed for detailed abnormality in tissue architecture. According to the guidelines of the American Veterinary Medical Association (AVMA 2020), mice were euthanized by cervical dislocation and finally, animals were monitored and left in the chamber for at least 1 min after respiration had ceased. Moreover, the organs were weighed and processed accordingly for histopathological analysis.

#### 4.9.1. Acute Toxicity Studies

This study was done in accordance with NIH’s OECD-420 guidelines for the Testing of Chemicals. **1** and **JR-9** were tested for acute toxicity in female BALB/c mice (*n* = 5 per group). Mice were fasted overnight before dosing and were administered with 2000 mg/kg as a single dose via oral gavage whereas normal saline (0.9%) to the control groups [55,56]. The animals were continuously observed for any changes for 30 min, 1, 2, 4 and 24 h and thereafter once a day for the next 14 days. Daily measurement of feed and water intake was measured along with weekly body weight change, biochemical and hematological parameters.

#### 4.9.2. LPS Challenge and Serum Cytokine Detection

LPS (1 mg/kg; Sigma) was prepared in ice-cold, sterile phosphate-buffered saline (PBS) for intraperitoneal injection to mice with or without **1** and **JR-9**. The negative control, 0.9% saline and positive control, Dexamethasone (10 mg/kg) was used corresponding to the clinical dosage. Mice were euthanized 2 h after injection, and the blood samples were collected in BD. Vacutainer SST tubes from the retro-orbital plexus of mice for harvesting serum. Blood tubes were left to coagulate at ambient temperature for 15–30 min after collection. The samples were subjected to centrifuge at 10,000 rpm for 5 min. The top layer of serum was carefully transferred into a fresh 1.5 mL Eppendorf tube and stored at −80 °C until analysis.

#### 4.9.3. Analgesic Activity

Analgesic activity of **1** and **JR-9** was evaluated using the acetic acid-induced writhing test and tail immersion test in mice.

##### Acetic Acid Writhing Test

The procedure was demonstrated by Koster et al. in 1959 [57,58]. In this experiment, mice (*n* = 5 per group) were given a standard analgesic drug, diclofenac sodium (20 mg/kg, i.p.), **1** and **JR-9** (40, 20, 10, 5, 1 mg/kg orally), 30 min before intraperitoneal injection of 0.6% glacial acetic acid (10 mL/kg body weight). The number of writhes (a wave of abdominal muscle contractions followed by hind-limb extension is the hallmark of this condition) were counted from 5 min after acetic acid administration to 20 min after injection and represented as a percentage of protection. Analgesic properties were evidenced by a decrease in the number of writhes. The following formula was used to compute the percentage of protection against the writhing caused by acetic acid:

$$\%\;Protection=\frac{No.\;of\;writhing\;in\;control-No.\;of\;writhing\;in\;treated}{No.\;of\;writing\;in\;control}\times 100$$

##### Tail Immersion Test

The tail immersion test was also used to assess the analgesic activity of our compounds, as explained by Aydin [59,60]. Thirty minutes before and after intraperitoneal administration of the standard analgesic drug, diclofenac sodium (20 mg/kg, i.p.), and subcutaneous injection of **1** and **JR-9**, the response time was assessed after that every 30 min up to 120 min.

##### Carrageenan-Induced Paw Edema Test

Carrageenan-induced paw edema is a well-established model of acute inflammation for testing anti-inflammatory medicines in mice. The anti-inflammatory activity of **1** and **JR-9** was examined in mice with carrageenan-induced paw edema. Carrageenan (0.1 mL of 1% *w/v* solution) was injected subcutaneously under the plantar aponeurosis in the right hind paw of rats to cause acute inflammation. The inflammation was measured by using an electronic digital caliper before and after carrageenan injection at 1, 3, 6, 8, and 10 h. The percent inhibition of edema was analyzed quantitatively against a vehicle-treated control group. The difference between the volume at 0 h and the volume at 1 h indicates paw edema in the first hour after carrageenan administration. As a result, paw edema was determined at 0, 1, 3, 6, 8, and 10 h. After that, the percentage of paw edema inhibition was calculated using the formula:

$$\%\;inhibition\;of\;paw\;edema=\frac{Vc-Vt}{Vt}\times 100$$

#### 4.9.4. Clinical Pathology

The blood samples were obtained from the eye and collected in a tube coated with 10% heparin for hematology and from the heart for clinical chemistry testing after the treatment period. Total white blood cell (WBC), Red blood cell count (R.B.C.), hematocrit (HCT), mean corpuscular volume (MCV), platelet count (PLT), and differential leukocyte count were among the hematological parameters examined. Serum alkaline phosphatase (ALP), alanine aminotransferase (ALT), total bilirubin, blood creatinine, glucose, and serum aspartate aminotransferase (AST) were also measured.

#### 4.9.5. Tissue Collection and Histopathological Examination

All of the animals were euthanized by cervical dislocation after the experiment. The kidneys, liver, lungs, and paw were collected and stored in 10% neutral buffered formalin for at least 48 h prior to processing for histopathological examination. The fixed tissues were embedded in paraffin, sectioned to 4 to 5 μm thickness using a microtome, mounted on glass microscope slides, stained with eosin and hematoxylin, and viewed under light microscopy.

## 5. Conclusions

In conclusion, this study demonstrated that Arteannuin-B (**1**), and its spirocyclic-2-isoxazoline derivative (**JR-9**), display promising anti-inflammatory efficacy against NO, TNF-α and IL-6 with lower cytotoxicity toward RAW 264.7 cells via down-regulation of NF-κB/P38 MAPK signaling. In addition, we discovered that as compared to **1**, the **JR-9** effectively inhibits LPS-stimulated macrophage inflammatory responses and also proved to be antianalgesic/nociceptive for the treatment of inflammation with lower toxicity. Although further mechanistic studies on the molecular mechanisms of **1** and **JR-9** activity are needed, this study has demonstrated the anti-inflammatory effects in murine models and macrophages. Overall, the findings offer empirical support for the application of **JR-9** which could be promising and helpful for the prevention and treatment of inflammatory illnesses.

## Figures and Tables

**Table 1:** The binding affinity of **1** and **JR-9** was computed by Cyscore analysis, and the ADME properties were evaluated using SwissADME.

Compound Name	Docking Score		Cyscore (Binding Affinity)		ADME				
	Dimer-Site	DNA Site	Dimer-Site	DNA Site	Log Po/w	Log S	Log Kp	TPSA	Bioavailability Score
**1**	−4.64	−4.80	−2.34	−2.53	3.89	−5.42	−5.61 cm/s	60.42 Å²	0.55
**JR-9**	−4.65	−5.66	−2.54	−2.87	3.89	−5.42	−5.61 cm/s	60.42 Å²	0.55

Log Po/w: n-octanol/water partition coefficient; Log S: molar solubility in water; Log Kp: permeability coefficient; TPSA: Topological polar surface area.

**Table 2:** The effects on writhing by **1** and **JR-9** administered 30 min before intraperitoneal 0.6% acetic acid in mice. Each value represents mean ± SD or percentage inhibition of pain as compared to control animals, *n* = 5 (**** *p* < 0.0001, ** *p* < 0.001 * *p* < 0.05 indicated significant difference with the control group assessed by one-way ANOVA followed by Dunnett’s test).

Group	Dose (mg/kg)	No. of Writhes	% Inhibition
Control	0.2	--	--
Diclofenac Sod. (ip)	20	15.09 ± 0.53 ****	40.42
1	40	11.89 ± 0.68 ****	53.03
	20	14.25 ± 1.91 ****	43.74
	10	19.25 ± 0.31 ****	23.97
	5	22.55 ± 1.58 **	10.94
	1	23.43 ± 0.16 *	07.48
JR-9	40	10.07 ± 0.37 ****	60.21
	20	11.83 ± 0.89 ****	53.29
	10	16.95 ± 0.39 ****	33.05
	5	19.5 ± 0.14 ****	23.01
	1	23.24 ± 0.63 *	8.25

**Table 3:** The effects of pre-treatment with **1** and **JR-9** at intervals of 30 min on tail withdrawal in rats whose tails were dipped in hot water (55.0 ± 0.5 °C). Each value represents mean ± SD, *n* = 5 (*p* < 0.0001 (indicated by a), *p* < 0.001(indicated by b) and *p* < 0.05 (indicated by c) indicated significant difference as compared to the control group assessed by two-way ANOVA followed by Tukey test).

Group	Dose (mg/kg)	0 min	30 min	60 min	90 min	120 min
Control		2.59 ± 0.14	2.41 ± 0.11	2.53 ± 0.11	2.40 ± 0.04	2.37 ± 0.06
Diclofenac Sod.	20	2.43 ± 0.11	5.27 ± 0.21 ^a^	6.49 ± 0.29 ^a^	7.50 ± 0.20 ^a^	7.84 ± 0.09 ^a^
1	40	2.70 ± 0.14	3.43 ± 0.15 ^a^	4.75 ± 0.24 ^a^	5.45 ± 0.08 ^a^	6.34 ± 0.21 ^a^
	20	2.47 ± 0.09	3.16 ± 0.15 ^a^	4.28 ± 0.8 ^a^	4.89 ± 0.11 ^a^	5.28 ± 0.36 ^a^
	10	2.55 ± 0.18	3.07 ± 0.14 ^b^	3.94 ± 1.32 ^a^	4.45 ± 0.06 ^a^	4.67 ± 0.19 ^a^
	5	2.61 ± 0.29	2.86 ± 0.05	3.55 ± 0.12 ^a^	3.66 ± 0.29 ^a^	4.15 ± 0.17 ^a^
	1	2.49 ± 0.08	2.57 ± 0.09	2.85 ± 0.06	2.91 ± 0.04 ^c^	3.69 ± 0.08 ^a^
JR-9	40	2.42 ± 0.12	5.21 ± 0.8 ^a^	5.35 ± 0.21 ^a^	5.70 ± 0.11 ^a^	6.44 ± 0.39 ^a^
	20	2.58 ± 0.14	4.5 ± 0.07 ^a^	4.61 ± 0.30 ^a^	5.2 ± 0.01 ^a^	5.65 ± 0.02 ^a^
	10	2.44 ± 0.10	4.42 ± 0.03 ^a^	4.54 ± 0.01 ^a^	5.08 ± 0.08 ^a^	5.43 ± 0.16 ^a^
	5	2.53 ± 0.30	4.19 ± 0.11 ^a^	4.27 ± 0.11 ^a^	4.34 ± 0.08 ^a^	4.62 ± 0.32 ^a^
	1	2.59 ± 0.16	3.44 ± 0.39 ^a^	3.43 ± 0.39 ^a^	3.84 ± 0.12 ^a^	3.91 ± 0.04 ^a^

**Table 4:** Effect of **1** and **JR-9** on biochemical and hematological parameters in Female BALB/c mice. Values are expressed as mean ± SD for five female mice in each group. RBC: red blood count; HCT: hematocrit; MCV: mean corpuscular volume; WBC: white blood cells; ALT: alanine aminotransferase; AST: aspartate aminotransferase; ALP: alkaline phosphatase.

S. No.	Parameters	Control	1		JR-9		Reference Ranges
			1 mg/kg	40 mg/kg	1 mg/kg	40 mg/kg	
1.	RBC (10^6^/µL)	10.3 ± 2.2	9.9 ± 0.9	11.2 ± 0.43	9.58 ± 1.15	12.18 ± 0.91	6.93–12.24
2.	HCT (%)	46.4 ± 1.5	43.91 ± 1.7	45.9 ± 1.34	46.43 ± 2.34	52.11± 1.49	42.1–68.3
3.	MCV (fL)	53.1 ± 1.9	54.5 ± 2.5	56.7 ± 0.43	61.92 ± 0.45	63.9 ± 1.4	50.7–64.4
4.	WBC (10^3^/µL)	4.49 ± 1.2	5.32 ± 0.28	7.64 ± 1.59	7.35 ± 0.38	9.79 ± 2.25	3.48–14.03
5.	Neutrophils (%)	10.8 ± 1.4	15.9 ± 0.73	17.42 ± 1.9	16.29 ± 3.75	24.42 ± 1.6	9.8–39.11
6.	Lymphocytes (%)	54.8 ± 0.5	55.91 ± 0.3	63.24± 2.8	64.35 ± 2.1	72.33 ± 2.81	48.81–83.19
7.	Monocytes (%)	6.34 ± 0.3	9.34 ± 0.95	7.38 ± 1.12	5.59 ± 2.39	9.42 ± 1.45	3.29–12.48
8.	Eosinophils (%)	0.4 ± 0.23	0.5 ± 0.11	2.3 ± 0.91	3.42 ± 0.52	4.15 ± 0.34	0–4.9
9.	Basophils (%)	0.2 ± 0.15	0.95 ± 0.7	1.4 ± 0.11	1.39 ± 0.34	1.45 ± 0.21	0–1.8
10.	Platelets (10^3^/µL)	91 ± 5.8	434 ± 13.4	632 ± 11.3	579 ± 14.38	714 ± 9.36	420–1698
11.	Glucose (mg/dL)	169 ± 7.1	174 ± 3.49	195 ± 2.54	159 ± 9.42	178 ± 7.68	129–329
12.	Creatinine (mg/dL)	0.41 ± 0.003	0.49 ± 0.01	0.39 ± 0.06	0.26 ± 0.07	0.38 ± 0.12	0.2–0.4
13.	Total Bilirubin (mg/dL)	0.3 ± 0.001	0.4 ± 0.04	0.36 ± 0.02	0.42 ± 0.02	0.46 ± 0.06	0.2–0.5
14.	A.L.T. (U/I)	59.4 ± 3.1	69.8 ± 4.4	78.32 ± 15.1	75.49 ± 7.45	97.36 ± 4.39	41–131
15.	A.S.T. (U/I)	65 ± 10.9	74.5 ± 5.21	83.98 ± 3.4	79.21 ± 6.94	95.91 ± 2.91	55–352
16.	A.L.P. (U/I)	221 ± 12.9	215.7 ± 14.3	239 ± 22.6	229.9 ± 17.3	245.3 ± 11.2	118–433

## Data Availability

The data used to support the findings of this study are available from the corresponding author upon request. The synthesis method and purification profiling is available in Reference [39].
